# Physical Mobility Impairment and Risk for Cardiovascular Disease

**DOI:** 10.1089/heq.2019.0065

**Published:** 2019-10-24

**Authors:** Mary L. Wilby

**Affiliations:** School of Nursing and Health Sciences, La Salle University, Philadelphia, Pennsylvania.

**Keywords:** cardiovascular disease, disability, mobility impairment

## Abstract

**Background:** People with disabilities (PWD) often self-report reduced access to preventive health services and poorer health than people without disability. Risk factors for chronic disease are more prevalent in PWD, increasing risk for secondary conditions including cardiovascular disease (CVD).

**Methods:** Logistic regression was used to analyze data from the 2016 Behavioral Risk Factor Surveillance Survey to explore the relationship between disability with mobility impairment and CVD.

**Results:** Difficulty walking and climbing stairs significantly predicted concomitant CVD and diabetes in logistic regression models.

**Conclusion:** Information from this study may be useful in addressing CVD risk for adults with mobility impairments.

## Background

The Americans with Disabilities Act was enacted in 1990 to eliminate discrimination against those with disabilities in all public places including employment, transportation, government services, and education, granting people with disabilities (PWD) the same rights as nondisabled citizens.^1^

Disability has traditionally been defined by a medical model that classifies it as a structural or functional impairment that limits activity. More recently, integrative models have included environmental barriers, negative attitudes regarding disability, structural barriers, and stigma as factors influencing disability. Functional impairment is one factor driving disability, but structural barriers, stigma, and negative attitudes regarding disability reduce access to health care and participation in health-promoting activities. PWD may have difficulty obtaining assistive devices, such as motorized wheelchairs and limb prostheses necessary to allow mobility to access services because of insurance regulations and other bureaucratic barriers.^2^

PWD frequently self-report poor health and experience poorer health outcomes than nondisabled people.^3–9^ There are multiple challenges for PWD with mobility impairment seeking health promotion and health maintenance services including transportation, access to sites where services are delivered, and providers who are insensitive or unaware of the accommodations needed to allow PWD to utilize services. Barriers contribute to the lack of participation of PWD in many preventive services, delays in treatment, and unmet health care needs.^5–9^

Nearly 20% of U.S. adults experience some form of disability. The incidence of disability is expected to rise with increased survival rates of children and young adults with previously fatal conditions.^9,10^ Individuals with chronic disabling conditions have added risk for secondary conditions, which occur as a result of the primary disabling condition, causing pathology, impairment, functional limitation, or additional disability.^10,11^

More than 60% of U.S. adults with two chronic health conditions are younger than 65 years old. People <65 years with multiple comorbid conditions (MCC) are more likely to report disability and modifiable risk factors for cardiovascular disease (CVD) including obesity and smoking.^12^ Costs of care for individuals with MCC are expected to accumulate over time adding significant financial burden to the health care system.^2^ Diabetes mellitus (DM) and obesity are among a number of chronic diseases increasing in prevalence in the United States with both conditions strongly associated with the risk for development of CVD. The presence of these conditions coinciding with disability can have significant consequences for long-term health in PWD.^7^

Little is known about the prevalence and predictors of chronic comorbid conditions in people with long-term disabilities. People with developmental and intellectual disabilities often experience physical conditions that impair physical mobility including gait disorders, musculoskeletal deformities and pain, and paralysis.^11,13^ PWD are more likely to report chronic diseases unrelated to their disability, including DM, CVD, stroke, and cancer.^7^ Rasch et al. identified a higher prevalence of comorbid conditions in adults with mobility limitations than in those without mobility impairment as well as an increased frequency of combinations of risk factors for CVD including obesity, diabetes, and hypertension (HTN).^10,11^

Increased risk of CVD and stroke among individuals with spinal cord injury has been identified with risk increasing with age.^13^ Some modifiable risk factors for CVD are more prevalent in PWD. In a study using data from the National College Health Assessment, cigarette smoking prevalence in college students with disabilities was significantly higher than in those without disabilities.^14^

In a longitudinal self-report survey involving 1594 individuals conducted to learn more about the incidence and age of onset of chronic comorbid conditions in people with long-term physical disabilities, arthritis, CVD, HTN, DM, and cancer, as well as behaviors including alcohol and tobacco use and physical inactivity were reported. Individuals between the ages of 56 and 65 years were more likely to report development of these chronic conditions. Conditions most prevalent included HTN, arthritis, and cancer. All conditions were more prevalent in those who already had pre-existing chronic medical conditions.^15^

In a study using National Health Interview Survey data, individuals with lifelong disabilities had significantly higher odds of all chronic conditions than people without limitations with the largest effect in CVD.^16^ Some risk factors for these conditions, including overweight and obesity, increased waist circumference, and smoking, are modifiable and could be amenable to health promotion interventions. It has been demonstrated that even modest levels of physical activity can offer benefits in ameliorating CVD risk in adults of all ages.^17,18^ The ability to engage in activities such as walking and cycling is associated with favorable CVD risk profiles.^19^ Staff-supported and self-directed exercise programs have demonstrated that PWD accept increased aerobic exercise activity as well as increased socialization.^20^

In a study in the Netherlands, people over the age of 55 years with disability in activities of daily living and mobility had a 10-year shorter life expectancy than nondisabled people.^21^ Obesity and overweight were noted to be the same or higher among a group with developmental disabilities than in the general population with rates of HTN, hypercholesterolemia, CVD, and other chronic conditions being highest. Improvement in modifiable risk factors including inactivity and obesity has been demonstrated with developmentally disabled adults with structured exercise programs.^22,23^

Use of a wheelchair as the sole means of mobility has a significant effect on musculoskeletal and cardiovascular function. A high incidence of metabolic syndrome with increased blood pressure, blood sugar, abdominal obesity, and serum cholesterol has been noted in wheelchair users. Use of upper extremity muscles rather than larger lower extremity muscles uses less energy, making it more difficult for wheelchair users to maintain a healthy body mass index.^24^

CVD is the leading cause of death in the United States and risk factors, including obesity, DM, and physical inactivity, are significant contributors to its development. Impaired mobility in people with physical disabilities may contribute to development of predisposing factors and pose significant risk for development of DM and CVD in this population. For this reason, it is important to learn more about the relationship between impaired mobility and CVD and DM among PWD. The purpose of this project was to investigate two research questions:

(1) Does difficulty walking or climbing stairs predict cardiovascular disease?
(2) Does difficulty walking or climbing stairs predict diabetes?

## Methods

Secondary analysis of weighted data from the Behavioral Risk Factor Surveillance Survey (BRFSS) was utilized. The BRFSS is a primary source of data about health risk behaviors, chronic health conditions, and use of preventive services collected from adult U.S. residents through random digit dialed telephone interviews. Data are collected during >400,000 interviews per year.^25^ The Centers for Disease Control and Prevention (CDC) recommends use of weighted BRFSS data when generalizing to the population from the sample. Weighted BRFSS data have been adjusted to account for variation in participants' probability of selection, uneven selection of population subgroups relative to the population distribution, and nonresponses.^26^

Forward logistic regression was utilized to answer the research questions as the dependent variables were either dichotomous (question 1) or nominal (question 2) and the independent variable (difficulty walking or climbing stairs) was dichotomous. No interaction effects in the model were considered or included. The sample included valid data from 486,237 participants between 18 and 82 years with 210,606 males and 275,631 females.

## Results

To address question 1, logistic regression using CVD as the predicted variable and difficulty walking as the predictor variable indicated a high overall model fit (−2 log likelihood=204,901), which was statistically significant (*χ*^2^ [1, *n*=466,016]=12,464, *p*<0.001). Those with CVD were 99.6% more likely to have difficulty walking than those without CVD. The model reliably classified 93.8% of the cases and the calculated odds ratio (4.351) was significant (Table 1).

**Table 1.** Regression Coefficients

Prediction model	*β*	SE	Wald	df	*p*	Odds ratio
CVD	1.470	0.13	13,753.669	1	0.000	4.351
DM	1.331	0.009	22,135.698	1	0.000	3.785

This table illustrates the *β* weights, SEs, Wald values, df, *p* values, and odds ratios for two prediction models. In both models, difficulty walking/climbing stairs served as the independent predictor variable. The first row illustrates the first research question in which CVD was the predicted dependent variable. The second row illustrates the second research question in which DM was the predicted dependent variable.

CVD, cardiovascular disease; df, degrees of freedom; DM, diabetes mellitus; SE, standard error.

Forward logistic regression utilizing DM as the predicted variable and difficulty walking as predictor variable was used in answering question 2 and indicated a high overall model fit (−2 log likelihood=383,348) and was also statistically significant (*χ*^2^ [1, *n*=468,902]=20,587, *p*<0.001) result. People with DM were 64.9% more likely to have difficulty walking than nondiabetic people. The model correctly classified 84.5% of the cases. The calculated odds ratio (3.785) was significant (Table 1).

These results indicate a significant level of comorbidity with CVD and DM in BRFSS participants who reported mobility limitations, suggesting that decreased mobility is highly correlated with risk for both conditions as noted in other studies using large data bases.^8,9,16^

## Conclusions

The analysis of data from the BRFSS identifies statistically significant relationships between difficulty walking and climbing stairs and concomitant CVD and diabetes. A high proportion of Americans with mobility limitations have coexisting CVD and DM when compared with those without mobility limitation. It is likely that individuals with limited mobility have greater difficulty modifying risk factors when fitness facilities, health care providers, and other essential services are less accessible. This may interfere with the ability to engage in health-promoting activities, obtain preventive counseling and services, and delay modification of important risk factors through medical, nursing, and other health care provider-driven interventions.

When physical barriers interfere with access to essential services, it may be necessary to consider use of new and innovative strategies to provide them. Use of technology, including video conferencing, may allow PWD to participate in group education and counseling sessions, and virtual appointments with health care providers. Improved accessibility and transportation services to recreation facilities and increased availability of staff at facilities who recognize and are responsive to the accommodations needed or people with impaired physical mobility are also needed.

## Limitations

Although telephone interviews such as with the BRFSS offer the advantage of cost effectiveness, there are limitations. Telephone interviews involving large samples may inadvertently exclude some populations including those with low income and those in poor health. In addition, self-reported data may be less reliable as some individuals may underreport some information. In addition, the extent of physical mobility impairment is not fully explored in the BRFSS and the degree of impairment may be under or overestimated. Furthermore, there may be selection bias with some people who may screen telephone calls and not answer unsolicited calls.

It is evident that there is a relationship between mobility impairment and CVD. PWD face challenges in addressing modifiable CVD risk factors that are unique to this population. Information about the prevalence of risk factors and presence of CVD in people with long-term impaired mobility can be important for the development of programs aimed at reducing CVD and improving health in people with physical mobility impairments.

There is a need to address disease prevention and treatment among those with chronic health conditions and mobility impairment to promote health and improve quality of life. Specialized interventions that address the needs of wheelchair users and others with impaired mobility are needed to moderate risk. Health care professionals and policy makers alike have been reticent in giving adequate attention to the needs of PWD with regard to promoting access to services, assistive devices, and other necessities. It is not enough to provide services directed toward one's disability.

Health care providers and others able to influence policy must play a role in advancing a model of care for PWD that looks beyond day-to-day needs and limitations to one that emphasizes offering the potential for a healthier future with health promotion and disease prevention services integrated with tertiary care and rehabilitation.

## Abbreviations Used

BRFSS: Behavioral Risk Factor Surveillance Survey
CDC: Centers for Disease Control and Prevention
CVD: cardiovascular disease
df: degrees of freedom
DM: diabetes mellitus
HTN: hypertension
MCC: multiple comorbid conditions
PWD: people with disabilities
SE: standard error
